# The Effects of Exercise Training on Obesity-Induced Dysregulated Expression of Adipokines in White Adipose Tissue

**DOI:** 10.1155/2013/801743

**Published:** 2013-12-04

**Authors:** Takuya Sakurai, Junetsu Ogasawara, Takako Kizaki, Shogo Sato, Yoshinaga Ishibashi, Motoko Takahashi, Osamu Kobayashi, Shuji Oh-ishi, Junichi Nagasawa, Kazuto Takahashi, Hitoshi Ishida, Tetsuya Izawa, Hideki Ohno

**Affiliations:** ^1^Department of Molecular Predictive Medicine and Sport Science, Kyorin University, School of Medicine, 6-20-2 Shinkawa, Mitaka, Tokyo 181-8611, Japan; ^2^Department of Biochemistry, Sapporo Medical University School of Medicine, South-1 West-17, Chuo-ku, Sapporo, Hokkaido 060-8556, Japan; ^3^Department of Nursing, Kyorin University, Faculty of Health Science, 6-20-2 Shinkawa, Mitaka, Tokyo 181-8611, Japan; ^4^Department of Respiratory Medicine, Hachioji Medical Center, Tokyo Medical University, 1163 Tatemachi, Hachioji, Tokyo 193-0998, Japan; ^5^Department of Applied Physics and Chemistry, The University of Electro-Communications, 1-5-1 Chofugaoka, Chofu, Tokyo 182-8585, Japan; ^6^Third Department of Internal Medicine, Kyorin University, School of Medicine, 6-20-2 Shinkawa, Mitaka, Tokyo 181-8611, Japan; ^7^Department of Sports Biochemistry, Faculty of Health and Sport Science, Doshisha University, 1-3 Tatara Miyakodani, Kyotanabe, Kyoto 610-0394, Japan

## Abstract

Obesity is recognized as a risk factor for lifestyle-related diseases such as type 2 diabetes and cardiovascular disease. White adipose tissue (WAT) is not only a static storage site for energy; it is also a dynamic tissue that is actively involved in metabolic reactions and produces humoral factors, such as leptin and adiponectin, which are collectively referred to as adipokines. Additionally, because there is much evidence that obesity-induced inflammatory changes in WAT, which is caused by dysregulated expression of inflammation-related adipokines involving tumor necrosis factor-**α** and monocyte chemoattractant protein 1, contribute to the development of insulin resistance, WAT has attracted special attention as an organ that causes diabetes and other lifestyle-related diseases. Exercise training (TR) not only leads to a decrease in WAT mass but also attenuates obesity-induced dysregulated expression of the inflammation-related adipokines in WAT. Therefore, TR is widely used as a tool for preventing and improving lifestyle-related diseases. This review outlines the impact of TR on the expression and secretory response of adipokines in WAT.

## 1. Introduction

In recent years, obesity caused by the hypertrophy of white adipose tissue (WAT) has steadily increased worldwide, and has become a serious social problem [1]. In 2010, the Organization for Economic Cooperation and Development (OECD) released a report on the current state of obesity and the cost-effectiveness of preventive measures [2, 3]. That report states that obesity rates have risen in many countries, and that one in two individuals is either obese or overweight in about half of OECD countries. It is widely known that obesity is a risk factor for various “lifestyle-related diseases” such as type 2 diabetes and hypertension, and that obesity and diabetes cause increases in atherosclerotic disease. Therefore, there is an urgent need to establish strategies for the prevention and improvement of obesity and diabetes.

Epidemiological studies have shown that exercise is effective for preventing and improving obesity and diabetes [4, 5]. For example, a study by Helmrich et al. [6] followed 5,990 male graduates of the University of Pennsylvania over 14 years and found that the risk of developing diabetes is reduced by 6% for every 500 kcal increase in weekly exercise. Furthermore, a study that followed 21,271 male U.S. doctors over five years revealed that even a once-weekly bout of exercise at an intensity that is sufficient to cause sweating reduced the risk of developing diabetes [7]. In addition, results from a study that followed 87,253 female U.S. nurses over eight years showed that the group that exercised at least once a week at an intensity sufficient to cause sweating had a relative risk of developing diabetes of 0.84 compared with a group that exercised less than once a week [8].

Although WAT was once considered to be merely a site for energy storage, in recent years it has become better understood at the molecular level; for example, how WAT secretes physiologically active substances, collectively known as adipokines, and how obesity-induced dysregulated expression of adipokines in WAT causes insulin resistance, which is the pathogenesis of diabetes [9–11]. Therefore, WAT is considered to be one of the tissues that play a critical role in the onset of lifestyle-related diseases, and the reduction of excess WAT and the improvement of abnormal adipokine secretion are important strategies for the prevention and improvement of lifestyle-related diseases. Exercise training (TR) not only causes a loss of WAT mass, but can also influence the secretory response and expression of adipokines in WAT. This review outlines the impact of TR on the adipokines in WAT.

## 2. Adipokines and the Inflammatory Response of WAT

**Figure 1:**
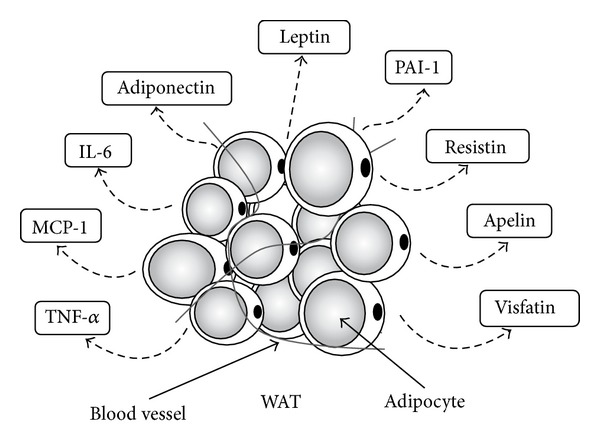
Adipokines secreted by white adipose tissue. White adipose tissue (WAT) secretes various humoral factors called adipokines. Adipokines have important effects on lipid and glucose metabolism, and so on. TNF-*α*, tumor necrosis factor-*α*; MCP-1, monocyte chemoattractant protein-1; IL-6, interleukin-6; PAI-1, plasminogen activator inhibitor-1.

The major role of subcutaneous and visceral WAT is to supply and store energy via adipocytes in WAT. Most of the ingested excess energy is stored within adipocytes in the form of triglycerides, which are formed through the binding of glycerol and fatty acids. During exercise, catchecolamines (adrenaline and noradrenaline) secreted from the adrenal medulla or the sympathetic nerve terminal break down triglycerides within adipocytes, and the resultant fatty acids are carried to skeletal muscle via the blood [12]. However, following the discovery of leptin by Zhang et al. [13] in 1994, leptin was established as a hormone that is secreted by WAT, and a string of new humoral factors that are secreted by WAT were discovered. Therefore, the old concept of WAT as a mere storage site for energy has been revised to also acknowledge it as an endocrine organ. The humoral factors secreted from WAT are collectively referred to as adipokines (Figure 1).

**Figure 2:**
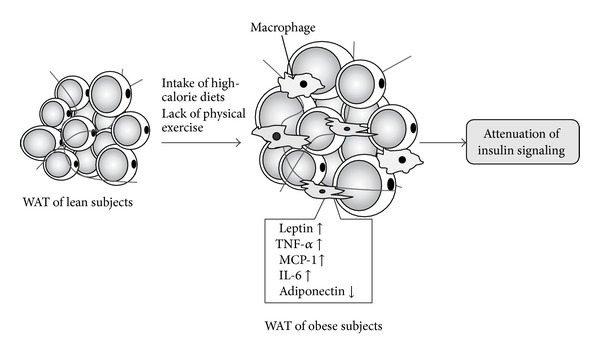
Model of the development of chronic inflammation in WAT. Adipocytes begin to grow as a result of factors such as excess energy intake and lack of exercise, and MCP-1 is secreted from these enlarged adipocytes. Macrophages infiltrate into WAT by the action of MCP-1, and as a result, increased expression of inflammatory adipokines (TNF-*α*, MCP-1, and IL-6) and decreased expression of anti-inflammatory adipokines (adiponectin) occur in WAT. Dysregulated expression of adipokines-induced inflammation of WAT contributes to the development of insulin resistance.

In recent years, it has become clear that obesity is a chronic and mild systemic inflammatory condition, and there is much evidence that chronic inflammation of WAT contributes to the development of insulin resistance. This systemic inflammation has become closely acknowledged as the molecular basis of diabetes [9–11]. When adipocyte hypertrophy occurs due to excessive energy intake or lack of exercise, infiltration by macrophages, which are one type of immunocompetent cell, is observed in WAT. In WAT infiltrated by macrophages, the production of proinflammatory adipokines, such as tumor necrosis factor-*α* (TNF-*α*) and monocyte chemoattractant protein 1 (MCP-1), is increased and the production of anti-inflammatory adiponectin is decreased, thereby causing chronic inflammation of WAT (Figure 2) [14–16]. This increase in proinflammatory adipokines is not limited to WAT, but also promotes insulin resistance in skeletal muscle and liver as a paracrine agent. Thus, the inflammatory response plays an important role in WAT activity.

## 3. Representative Adipokines and the Effects of TR

### 3.1. Leptin

Leptin is a hormone that acts on leptin receptors (ob-R) in the hypothalamus to strongly suppress appetite and promote increased energy expenditure [17–19]. There is strong ob-R expression in the arcuate nucleus, ventromedial hypothalamic nucleus, dorsomedial hypothalamic nucleus, and lateral hypothalamic area of the hypothalamus [18]. Although the expression of mRNA for leptin is elevated in the WAT of obese humans and animals and blood levels also increase, since there is impaired leptin action called “leptin resistance”, leptin does not function sufficiently to suppress appetite or promote energy expenditure [18, 20–22]. On the other hand, it is also known that leptin has inflammatory effects, such as increasing the expression of inflammatory cytokines involving TNF-*α* by acting on monocytes [9].

Evidence shows that seven weeks of spontaneous running TR reduces the expression of mRNA for leptin in the visceral and subcutaneous WAT of obese rats (Table 1) [23]. Additionally, other research indicates that even a short duration (four weeks) of spontaneous activity reduces leptin mRNA expression in rat WAT (Table 1) [24]. For obese humans, however, one study found that even 12 weeks of one-hour aerobic exercise sessions had no effect on the expression of mRNA for leptin in subcutaneous WAT (Table 1) [32]. On the other hand, there have been many studies on the effects of TR on the human blood levels of leptin (Table 2) [34–50]. Many cases have shown that concentrations of leptin decrease with a reduction in WAT mass (Table 2) [34, 36, 41–45, 47, 48]. By contrast, when no significant differences are observed in blood leptin levels after TR, neither is body fat reduced (Table 2) [34, 35, 39]. Therefore, the reduced blood concentration of leptin after TR is due more to the reduction in body fat caused by TR than to the effects of TR itself. Some studies, however, suggest that a longer duration (≥12 weeks) of TR or TR with caloric restriction can contribute to a reduction in blood leptin concentration that is independent of the influence of body fat reduction (Table 2) [34, 37, 40, 46].

Several studies have also concentrated on the effects of resistance training, such as the bench press exercise, on blood leptin levels (Table 2). One study on postmenopausal obese women found that after performing three days a week of resistance training using machines and restricting diet for 16 weeks, blood leptin levels were decreased compared with pretraining levels, but that resistance training alone had no effect on leptin [49]. However, when elderly individuals were divided into low intensity (45–50% 1 repetition maximum [RM]), moderate intensity (60–65% 1 RM), and high intensity (80–85% 1 RM) groups and performed 60-minute exercise sessions three times a week for six months, blood leptin levels were lower in all the groups compared with the respective pretraining levels, and the magnitude of this decrease was significantly greater in the high intensity group than in the low and moderate intensity groups [50]. Furthermore, although blood leptin levels were higher at six months after the end of training than immediately after the end of training, the levels remained significantly lower than their pretraining values in the high intensity group [50].

### 3.2. TNF-*α*

Since the discovery that gene expression of the major inflammatory cytokine TNF-*α* is elevated in WAT in animal models of obesity, there have been many studies on its involvement in insulin resistance and its other actions [51, 52]. Expression of TNF-*α* increases not only in the WAT of obese animals, but also in that of obese humans; that is, TNF-*α* has a strong positive correlation with body mass index (BMI) and blood insulin levels [53–55]. TNF-*α* weakens insulin signaling by insulin receptor substrate 1-mediated inhibition of insulin receptor tyrosine kinase activity in areas such as skeletal muscle and causes reduced expression of glucose transporters and adiponectin in adipocytes, which contributes to the development of insulin resistance [56–58].

There is no clear consensus regarding the effects of TR on TNF-*α* in WAT (Table 1). For example, increased expression of TNF-*α* in visceral WAT in mice that became obese after six weeks of consuming a high-fat diet can be suppressed by spontaneous running [25, 26]. Additionally, our studies showed that nine weeks of treadmill running decreased the TNF-*α* protein content of the rat WAT [27, 28], but some results have shown a contrasting increase after TR [24, 29, 30]. Studies regarding obese individuals have also examined the effects of TR on TNF-*α* expression in WAT. Although one study found that TNF-*α* expression in the subcutaneous WAT of severely obese male and female adults decreased after 15 weeks of performing TR, such as walking for five days a week and undergoing diet therapy; a conflicting study on obese adults found that there was no change in TNF-*α* mRNA expression in subcutaneous WAT even when weight or body fat decreased after 12 weeks of aerobic exercise [32, 33].

There are also conflicting results for the blood concentrations of TNF-*α* (Table 3) [33, 42, 45, 59–63]. A study on diabetic patients showed that although there is no change in blood TNF-*α* concentration after four weeks of dietary restrictions and walking TR in nonobese diabetic patients, the concentration decreased in obese patients [59]. Furthermore, when obese adult women exercised on a bicycle ergometer for 30 minutes a day, five days a week at 70% $\dot{\text{V}}{\text{O}}_{2}$ max for 12 weeks, decreases in blood concentrations of both TNF-*α* and soluble TNF receptor 2 were observed in both the women with insulin resistance and those without [60]. In another study, however, a 15-week combination of diet therapy and TR did not affect the TNF-*α* level in obese individuals [33]. In yet another study, 12 weeks of endurance TR actually increased the blood concentration of TNF-*α* in adult women [61].

### 3.3. MCP-1

MCP-1, which is identified as a monocyte chemotactic factor, shows increased expression in the WAT of obese mice, and elevated MCP-1 contributes to inflammatory changes by inducing macrophage infiltration into WAT via its receptor, C-C chemokine receptor-2, which is expressed in monocytes and macrophages [70, 71]. In mice that are genetically modified to only express MCP-1 excessively in adipocytes, infiltration into visceral WAT by macrophages is elevated when compared with control mice, and there is increased expression of macrophage markers and TNF-*α* genes in the tissue, as well as increased insulin resistance [70, 71]. Mice that consume a high-fat diet show increased expression of MCP-1 mRNA in visceral WAT, but this expression is suppressed by six weeks of spontaneous running activity (Table 1) [25]. Additionally, other studies where mice both consumed a high-fat diet and underwent treadmill running, MCP-1 mRNA expression, which had increased due to the mice's high-fat diet, was reduced by TR (Table 1) [26]. Moreover, nine weeks of treadmill running has reduced MCP-1 protein levels in rat subcutaneous and visceral WAT (Table 1) [27]. However, there were no changes in expression of mRNA for MCP-1 either in the subcutaneous and visceral WAT of rats that performed four weeks of spontaneous running or in the subcutaneous WAT of obese humans who performed 12 weeks of aerobic exercise (Table 1) [24, 32].

There seems to be consensus that TR diminishes blood levels of MCP-1 (Table 3). The blood concentration of MCP-1 was reduced in rats by nine weeks of treadmill running TR [27]. Studies on human patients with metabolic syndrome [64] and obese individuals [32] have also shown reductions and downward trends in MCP-1 after 12 weeks of TR. A 15-week combination of TR and diet therapy also reduced the blood concentration of MCP-1 in obese individuals [33].

### 3.4. Adiponectin

Adiponectin increases fatty acid oxidation and glucose uptake in skeletal muscle and inhibits gluconeogenesis in the liver [72, 73]. Adiponectin also inhibits the expression and secretion of TNF-*α* in macrophages and increases the production of anti-inflammatory cytokines such as interleukin (IL)-10 [74]. Therefore, adiponectin is thought to have anti-inflammatory effects. In accordance with that function, the expression of mRNA for adiponectin is reduced in the WAT of genetically obese mice and obese humans, and both obese individuals and diabetic patients have a lower blood concentration compared with healthy individuals [75, 76]. Insulin resistance and hypertension are improved when KKAy mice (mouse models of obesity and diabetes) are administered physiological concentrations of adiponectin, and insulin resistance is observed in KO mice deficient in adiponectin, suggesting that obesity-induced decreases in adiponectin expression in WAT are closely associated with the development of insulin resistance and the onset of diabetes [72, 73].

A 15-week combination of TR and diet therapy or 12 weeks of aerobic exercise has shown increases in the expression of mRNA for adiponectin in the subcutaneous WAT of obese individuals (Table 1) [32, 33]. In studies on rats, nine weeks of treadmill running has increased the mRNA expression in visceral and subcutaneous adipocytes (Table 1) [31]. In at least one study, short periods of consuming a high-fat diet increased adiponectin expression in the subcutaneous WAT of rats, and TR by spontaneous running activity suppressed this increase. That study found no effect of TR on adiponectin mRNA expression in visceral WAT (Table 1) [24].

As with leptin, there have been many studies on the effects of TR on the blood levels of adiponectin (Table 4) [32, 33, 38, 42, 45, 50, 65–69]. Although most indicate that there is no change, some studies show that it increases, so there is no consensus on this point. Hulver et al. [69] found that the blood concentration of adiponectin did not change after obese adults performed aerobic exercises such as running at 65–85% $\dot{\text{V}}{\text{O}}_{2}$ max four times a week over a period of six months. Another study on diabetic men also found no change in the blood concentration of adiponectin after eight weeks of performing aerobic exercise three times a week, even though the amount of visceral fat decreased [38]. Even after elderly obese men and women performed TR for 60 minutes on a treadmill or bicycle ergometer at 80–85% of their maximum heart rate five times a week for 12 weeks, there was no change in the blood concentration of adiponectin despite the deceases in BMI and body fat [67]. Contrary studies have found that 60 minutes of TR, such as running performed four times a week for four weeks, has led to increases in the blood concentration of adiponectin along with decreases in body fat in diabetics and individuals presenting impaired glucose tolerance [65]. In a similar manner, the blood concentration of adiponectin has been increased along with reduced BMI and body fat mass after seven months of TR such as slope jogging and dumbbells performed four to five times a week in obese young women [45].

### 3.5. IL-6

IL-6 is a cytokine that has a variety of functions such as regulating hematopoiesis, immune response, and inflammatory response. This cytokine also is known to have anti-inflammatory effects, and may have both proinflammatory and anti-inflammatory properties [77, 78]. Diabetic and obese individuals have high blood concentrations of IL-6, and its mRNA expression is elevated in the subcutaneous adipocytes of individuals presenting insulin resistance. Furthermore, IL-6 acts on adipocytes to inhibit insulin signaling [79, 80].

Many studies show that IL-6 levels increase in response to acute exercise; for instance, a single bout of exercise has increased the blood concentration of IL-6 more than 100 times. However, this increase in blood concentration was not due to increased production by WAT, but rather by increased production in skeletal muscle, an organ that produces IL-6 [78, 81]. A 15-week combination of TR and diet therapy reduces the expression of mRNA for IL-6 in the subcutaneous WAT of obese individuals (Table 1) [33]. However, although some studies show that the blood concentration of IL-6 decreases after TR, other studies have shown no change, so yet again there is no consensus (Table 5) [33, 42, 62, 63, 66, 68, 78].

## 4. The Relationship between TR-Induced Changes in Adipokine Expression and WAT Mass

**Figure 3:**
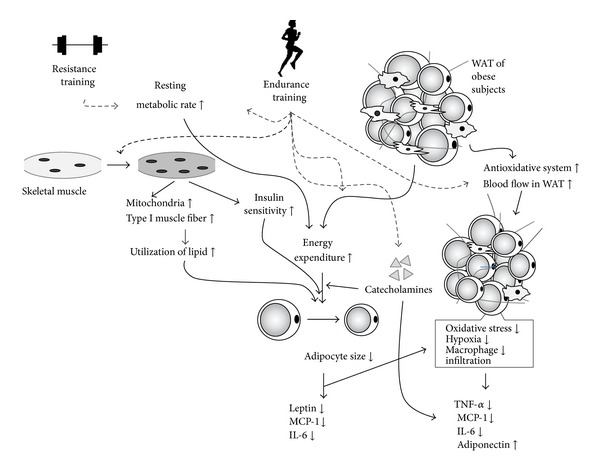
Schematic model for the effects of exercise training on expression of adipokines in WAT. During endurance training, type I muscle fibers in skeletal muscle are selectively used for the execution of exercises, and therefore, energy expenditure using lipid increases. Triglycerides within the adipocytes are broken down due to the secretion of catecholamines, and the resultant fatty acids are transported to tissues such as skeletal muscle. When exercise is repeated, adipocyte size is lessened. Decreases in adipocyte size are considered to result in the attenuation of dysregulated expression of adipocyte size-sensitive adipokines, such as leptin and oxidative stress in WAT. Moreover, catecholamine itself seems to correct disarray of adiponectin and TNF-*α* in WAT of obese subjects. In addition, endurance training might suppress oxidative stress and a hypoxic state of WAT due to an enhanced antioxidative system and increases in blood flow, respectively, which lead to the attenuation of the dysregulated expression of inflammatory-related adipokines involving TNF-*α* and MCP-1. In skeletal muscle, endurance training produces transition to type I muscle fiber following the increase in mitochondria biogenesis and enhances insulin sensitivity. Consequently, enhanced glucose/lipid metabolism in skeletal muscle decreases adipocyte size. On the other hand, resistance and endurance training enhance resting metabolic rate, which is likely to cause the alteration of adipokine expression following WAT mass reduction due to increased energy expenditure in the resting state.

The size of WAT (adipocytes) greatly affects the expression of adipokines. As for leptin, mRNA expression and secretion are positively correlated with the size of adipocytes isolated from rodents and humans [31, 82–84]. Similarly, in isolated adipocytes of humans, secretion of TNF-*α*, MCP-1, and IL-6 is positively correlated with cell size, and after correction for the cell surface, there is still a significant difference between very large and small adipocytes for MCP-1 and IL-6 [83]. Nevertheless, mRNA levels for TNF-*α* show no significant correlation with mouse adipocyte volume [84]. On the other hand, although the expression of adiponectin is reduced in the WAT of genetically obese mice and obese humans, the mRNA expression and secretion of adiponectin is positively correlated with isolated adipocyte size in rats and humans [31, 75, 76, 83]. One of the reasons for this discrepancy is speculated that reduced adiponectin expression *in vivo* may be the result of inflammatory adipokines, such as TNF-*α*, rather than increases in the size of adipocytes [58]. It is well known that TR reduces WAT mass, and, therefore, the reduction of WAT is thought to be a major factor in the effects of TR on adipokine expression in WAT (Figure 3). However, further research is needed regarding other effects of TR. Recently, an interesting study has examined the relationship between TR-induced changes in adipokine expression and WAT mass. Christiansen et al. [32] divided obese subjects into a group that underwent 12 weeks of combined aerobic exercise and diet therapy and a group that underwent diet therapy only, and after adjusting weight loss to approximate amounts, found no difference in changes in either the expression of inflammatory-related adipokines in subcutaneous WAT or in the circulating markers of inflammation; that is, TR seemed to have had no weight-independent effects in that study. On the other hand, when the authors observed a reduced level of leptin mRNA and an elevated mRNA level of adiponectin in rat visceral adipocytes after nine weeks of treadmill running, it suggested that the decrease in leptin mRNA expression depended on a reduction in adipocyte size, and that the increase in adiponectin mRNA was mediated by factor(s) other than adipocyte size [31]. In addition, Oberbach et al. [66] found that actual increases in blood adiponectin after TR were of a higher magnitude than increases in blood adiponectin levels that were predicted according to a regression line drawn from the negative correlation between body fat and the blood concentration of adiponectin.

During exercise, the secretion of catecholamines from the adrenal medulla and sympathetic nerve peripheries breaks down triglycerides within the adipocytes [12]. Several reports have indicated that *β*-adrenoceptor agonists affect the expression of some adipokines, such as TNF-*α* and adiponectin in WAT. Administration of *β*-adrenoceptor agonists in lean mice results in upregulation of TNF-*α* and downregulation of adiponectin in epididymal WAT [85, 86]. These findings seem to conflict with the beneficial effects of exercise on the disturbance of adipokines. Nevertheless, during exercise, since energy consumption is enhanced, the blockage of lipogenesis by the impaired insulin signaling in WAT might play reasonable roles in the proper execution of exercise. In contrast to lean mice, *β*-adrenoceptor agonists recovered the declined mRNA expression of adiponectin and suppressed the overexpressed mRNA level of TNF-*α* in WAT of KKAy mice [87]. Therefore, in obese and type 2 diabetic patients, it is likely that the secretion of catecholamines during exercise is one of the reasons for the attenuation of dysregulated adipokine expression in WAT (Figure 3).

Various possible mechanisms besides decreased WAT mass and secretion of catecholamines have been proposed, including decreased oxidative stress and improvement of hypoxia in WAT (Figure 3). Adipocytes have produced reactive oxygen, and obesity-induced increases in oxidative stress in WAT may be a cause of the dysregulated expression of inflammatory-related adipokines [88]. Studies have shown significantly lower levels of lipid peroxidation in WAT around the epididymis and retroperitoneum of rats that had undergone TR compared with a control group, and elevated protein levels of the antioxidant enzyme manganese superoxide dismutase (Mn-SOD) in the epididymal WAT of TR group rats [27, 28]. In that study, not only were protein levels of TNF-*α* and MCP-1 significantly lower in the epididymal WAT of the TR group, compared with those of the control group, the phosphorylation of extracellular signal-regulated kinase, which is activated by reactive oxygen and is important for the expression of MCP-1, also was reduced by TR in WAT around the epididymis and retroperitoneum [27, 89]. TR reduced WAT mass, which likely contributed to decreased oxidative stress in WAT. Nevertheless, because acute exercise elevates oxidative stress in the body [90], the adaptation of WAT against exposure to oxidative stress from exercise, in other words, the expansion of antioxidant systems via increases in Mn-SOD, could be one reason for decreased levels of proinflammatory adipokines.

Recent evidence that tissue hypoxia is involved in obesity-induced inflammatory changes in WAT has attracted the attention of researchers. In fact, oxygen partial pressure is lower in the WAT of obese animals and humans compared with controls, and results show that this may be related to the inflammatory response in WAT [91, 92]. Although some studies have focused on the impact of TR on blood flow in WAT, results from those studies appear to indicate that when WAT mass decreases due to TR, blood flow in the tissue increases [93]. We found that expression of mRNA for vascular endothelial growth factor and its receptor was elevated in the WAT stromal vascular fraction cells of rats that had engaged in TR, and that the vascular endothelial cell count per unit area had increased [94]. Thus, the increased blood flow to WAT produced by TR eliminated the obesity-induced hypoxia in WAT and could possibly have led to a weakening of the inflammatory changes in WAT.

## 5. Can TR That Does Not Alter Body and WAT Mass Alleviate Dysregulated Expression of Adipokines?

In many of the previous studies that examined the effects of TR on adipokine expression in WAT and on the blood levels of adipokines in human subjects, body mass, BMI, or WAT mass reduction is observed (Tables 1–5). For this reason, it remains unknown whether or not low-intensity TR that does not entail such reduction alters adipokine expression in WAT or blood adipokine levels. As described in the previous chapter, adipokine expression is affected by the size of the WAT (adipocyte). Among adipokines, expression of leptin seems to be especially largely affected by adipocyte size [82–84]. In studies that examined the effect of TR on the blood leptin level in human subjects, results indicated that, in many cases, blood leptin levels do not change without a reduction in body fat; that is, decreased blood leptin levels are thought to be caused by exercise-induced WAT mass reduction (Table 2) [34, 36, 41–45, 47]. In contrast, some studies have reported that the reduced blood leptin level shows beneficial effects of TR without WAT mass reduction. For example, studies on adult males and females have shown that only the female subjects exhibit reduced blood leptin levels without body fat loss after undergoing 12 weeks of TR [40]. Similarly, Ishii et al. [37] have demonstrated that TR in type 2 diabetic subjects reduces serum leptin levels independent of changes in body fat mass. On the other hand, increased blood adiponectin level through TR is also accompanied by reductions in body mass, BMI, or WAT mass (Table 4) [33, 45, 48, 65, 66]. Although Hsieh and Wang [48] observed that the blood adiponectin level was significantly elevated in type 2 diabetes patients who performed low-intensity TR (20 min/day, 50–74% maximum heart rate) and adequate calorie restriction for one year, this particular study showed that body mass reduction seemed to be beneficial for increases in adiponetin. However, other reports indicate that blood adiponectin levels do not change if body mass is decreased [38, 42, 67]. Thus, it is difficult to conclude at this stage whether loss of body and/or WAT mass is indispensable for adiponectin elevation. Moreover, the effects on TR-induced body and WAT mass reduction may differ depending on the type of adipokine. Taken together, these results show that although further examination is necessary, it is conceivable that changes in adipokine expression in WAT and blood adipokine level require TR that is sufficiently intense to reduce body mass or more specifically WAT mass.

## 6. Changes in Skeletal Muscle through TR and Its Impact on Expression of Adipokines in WAT

Skeletal muscle is responsible for physical exercise, and it is the largest tissue in the body. Undernutrition, aging, and sickness cause a decline in skeletal muscle mass (a condition known as muscular atrophy), deteriorating one's exercise capacity [95, 96]. Moreover, skeletal muscle has a substantial impact on the overall metabolism of the body. For instance, skeletal muscles in patients with obesity and type 2 diabetes have reduced glucose metabolic capacity due to insulin resistance [97], and these observations are considered to be associated with the patients' clinical conditions. Many studies have shown that TR can increase mitochondrial proliferation and boost the expression of a glucose transporter 4 (GLUT4), and can in turn enhance lipid and glucose metabolic capacities [98–100]. Among the molecules involved in exercise-induced enhancement of glucose/lipid metabolic capacity in skeletal muscle, AMP-activated protein kinase (AMPK) and peroxisome proliferator-activated receptor-*γ* coactivator-1*α* (PGC-1*α*) have been gaining a great deal of attention.

AMPK is an enzyme that is activated when ATP is converted to AMP and is a sensor of energy status that maintains cellular energy homeostasis [101, 102]. Skeletal muscle AMPK is activated by muscle contraction [103], treadmill running [104], and stimulation by its agonist aminoimidazole carboxamide ribonucleotide (AICAR) [105]. Upon activation, AMPK induces the phosphorylation of downstream effectors to elevate glucose uptake. Glucose uptake elevation has been associated with the induction of GLUT4 translocation to the cell membrane [106–108]. AMPK activity has also been reported to be involved in fatty acid uptake through the fatty acid translocase FAT/CD36 and fatty acid oxidation mediated by reduced acetyl-CoA carboxylase enzymatic activity [103, 109]. TR has been shown to enhance both expression and activation of AMPK in skeletal muscle, and chronic AMPK activation in skeletal muscle can increase the number of mitochondria even in the absence of TR, suggesting that TR-induced AMPK activation is strongly involved in the increase in the mitochondria of skeletal muscle [110, 111]. However, a conclusion is yet to be drawn because AMPK KO mice that underwent TR also showed increases in skeletal muscle mitochondria [112].

Transcription coactivator PGC-1*α* forms a complex with nuclear receptors and transcription factors to regulate gene transcriptions, or more specifically, expression of genes involved in mitochondrial biosynthesis [113–115]. In fact, mice with PGC-1*α* overexpression showed (1) increased number of mitochondria, (2) enhanced expressions of oxidizing enzymes such as cytochrome oxidase in skeletal muscle, and (3) transition to type I muscle fibers [114, 115]. Physical exercise increases PGC-1*α* transcription and potentially PGC-1*α* activity through posttranslational modifications, and concomitant PGC-1*α*-mediated gene regulation is suggested to be an underlying mechanism for adaptations in skeletal muscle, when exercise is repeated [115].

Muscle consumes the most energy out of all tissues in the body. Therefore, increases in mitochondria and increased insulin sensitivity in skeletal muscle by endurance TR are thought to dramatically impact the energy consumption of the whole body. Moreover, enhanced glucose/lipid metabolism in skeletal muscle is considered to be indirectly involved in WAT reduction, which results in altered adipokine expression (Figure 3). Additionally, because resting metabolic rate (RMR), which is the largest component of the daily energy budget in most human societies, is reportedly elevated owing to both aerobic and resistance training in human subjects, although some studies have failed to find such an effect [116], enhanced RMR is likely to cause alteration of adipokine expression following WAT mass reduction due to increased energy expenditure in the resting state (Figure 3). Nevertheless, the detailed mechanisms and whether mediators, such as myokines, from skeletal muscle act on the existence of WAT remain unknown. On the other hand, it is interesting that evidence is mounting on the new effects of adipokine on skeletal muscle metabolic capacity. Recent observation of KO mice showed that a lack of adiponectin receptor in their skeletal muscle showed a reduced mitochondrial content, reduced type I muscle fibers, and decreased capacity for exercise, suggesting that adiponectin is involved in mitochondrial biogenesis in skeletal muscles [117]. Furthermore, there is a significant positive correlation between blood adiponectin level and AMPK activity in the lateral great muscles in men [118]. In the future, it is crucial to examine the effect of TR on adipokine expression not only in WAT alone but also in terms of cross-talk between WAT and other tissues involving skeletal muscle. Further investigations are warranted.

## 7. Conclusions

Although reports on the effects of exercise on adipokine levels in WAT and blood may not always agree due to differences in experimental subjects, exercise intensity, or exercise duration, it is reasonable to believe that there is at least a positive effect. Although TR-induced WAT reduction is one of the key reasons for attenuation of dysregulated expression of adipokines, detailed studies about not only WAT-reducing effects of TR but also other effects, such as antioxidative effects and angiogenic effects, will be necessary to show the usefulness and distinctiveness of TR. Furthermore, it may be significantly beneficial to examine the cross-talk between WAT and other tissues involving skeletal muscle and to what degree WAT contributes to TR-induced changes in blood adipokine levels. Because the importance of exercise as a tool for preventing and improving obesity and lifestyle-related diseases can be expected to grow in the future, further research is desirable.

## Figures and Tables

**Table:** (a) Animal studies

Citation	Experimental animals	Exercise program	Diet restriction	Duration of intervention	WAT used in experiment	Effects of TR on expression of adipokines in WAT	Changes of body mass (BM), body mass index (BMI), fat mass (FM), and % body fat mass (% BFM)
Zachwieja et al. [23]	Diet-induced obesity sensitive rats	Voluntary wheel running	None	7 weeks	Epididymal and inguinal WAT	**Epididymal WAT** Leptin mRNA: decrease (*P* < 0.05) **Inguinal WAT** Leptin mRNA: decreasing trend	Epididymal and inguinal FM: decrease
	Diet-induced obesity resistant rats	Voluntary wheel running	None	7 weeks	Epididymal and inguinal WAT	**Epididymal WAT** Leptin mRNA: decrease (*P* < 0.05) **Inguinal WAT** Leptin mRNA: decreasing trend	Epididymal and inguinal FM: decrease

Gollisch et al. [24]	Rats chow diet	Voluntary wheel running	None	4 weeks	Visceral and subcutaneous WAT	**Visceral WAT** Leptin mRNA: decrease (*P* < 0.05) TNF-*α* mRNA and protein: NS MCP-1 mRNA: NS Adiponectin mRNA: NS IL-6 mRNA: NS **Subcutaneous WAT** Leptin mRNA: NS TNF-*α* mRNA and protein: increase (*P* < 0.05) MCP-1 mRNA: NS Adiponectin mRNA: NS IL-6 mRNA: increase (*P* < 0.05)	BM: NS; Visceral and subcutaneous FM: decrease
	Rats HFD	Voluntary wheel running	None	4 weeks	Visceral and subcutaneous WAT	**Visceral WAT** Leptin mRNA: decrease (*P* < 0.05) TNF-*α* mRNA and protein: NS MCP-1 mRNA: NS Adiponectin mRNA: NS IL-6 mRNA: NS **Subcutaneous WAT** Leptin mRNA: decrease (*P* < 0.05) TNF-*α* mRNA: increase (*P* < 0.05) TNF-*α* protein: NS MCP-1 mRNA: NS Adiponectin mRNA: decrease (*P* < 0.01) IL-6 mRNA: increase (*P* < 0.05)	BM: decrease; Visceral and subcutaneous FM: decrease

Bradley et al. [25]	Mice chow diet	Voluntary wheel running	None	10 weeks (exercise: 6 weeks)	Perigonadal and mesenteric WAT	**Perigonadal WAT** TNF-*α* mRNA: decrease (*P* < 0.05) MCP-1 mRNA: decrease (*P* < 0.05) **Mesenteric WAT** MCP-1 mRNA: decrease (*P* < 0.05)	BM and FM: decrease
	Mice HFD	Voluntary wheel running	None	10 weeks (exercise: 6 weeks)	Perigonadal and mesenteric WAT	**Perigonadal WAT** TNF-*α* mRNA: decrease (*P* < 0.05) MCP-1 mRNA: decrease (*P* < 0.05) **Mesenteric WAT** MCP-1 mRNA: decrease (*P* < 0.05)	BM and FM: decrease

Vieira et al. [26]	Mice HFD	Treadmill running for 40 min/day on 5 times/week at 65–70% $\dot{\text{V}}$O_2_ max	None	18 weeks (exercise: 6 or 12 weeks)	Epididymal and retroperitoneal WAT	**Exercise for 6 weeks** Leptin mRNA: decreasing trend TNF-*α* mRNA: NS MCP-1 mRNA: NS **Exercise for 12 weeks** Leptin mRNA: decreasing trend TNF-*α* mRNA: decrease (*P* < 0.001) MCP-1 mRNA: decrease (*P* < 0.001)	**Exercise for 6 weeks** BM and epididymal FM: decrease **Exercise for 12 weeks** BM and epididymal FM: decrease

Sakurai et al. [27]	Rats chow diet	Treadmill running on 5 times/week. On the first day of training, all rats ran for 30 min at 15 m/min, and then running time and velocity were extended until rats were running for 90 min at 30 m/min.	None	9 weeks	Epididymal WAT	TNF-*α* protein: decrease (*P* < 0.05)	BM and epididymal FM: decrease

Sakurai et al. [28]	Rats chow diet	Treadmill running on 5 times/week. On the first day of training, all rats ran for 30 min at 15 m/min, and then running time and velocity were extended until rats were running for 90 min at 30 m/min.	None	9 weeks	Epididymal, retroperitoneal, and subcutaneous WAT	**Epididymal adipocyte** TNF-*α* mRNA: decrease (*P* < 0.05) MCP-1 mRNA: decrease (*P* < 0.05) **Epididymal WAT** TNF-*α* protein: decrease (*P* < 0.05) MCP-1 protein: decrease (*P* < 0.05) **Retroperitoneal WAT** TNF-*α* protein: decreasing trend MCP-1 protein: decrease (*P* < 0.05) **Subcutaneous WAT** TNF-*α* protein: NS MCP-1 protein: decrease (*P* < 0.05)	BM: decrease epididymal, retroperitoneal, and subcutaneous % BFM: decrease

Lira et al. [29]	Rats chow diet	Treadmill running on 5 times/week at 55–65% $\dot{\text{V}}$O_2_ max. On the first day of training, all rats ran for 30 min. On the subsequent days of training, running time was extended 10 min each day until rats were running 60 min/day.	None	9 weeks	Retroperitoneal and mesenteric WAT	**Retroperitoneal WAT** TNF-*α* protein: NS **Mesenteric WAT** TNF-*α* protein: increase (*P* < 0.05)	BM and retroperitoneal FM: decrease Mesenteric FM: NS

Nara et al. [30]	Rats high-sucrose diet	Voluntary wheel running	None	4 and 12 weeks	Mesenteric and subcutaneous WAT	**Mesenteric WAT** **Exercise for 4 and 12 weeks** TNF-*α* mRNA: increase (*P* < 0.05) TNF-*α* protein: increase (*P* < 0.05) **Subcutaneous WAT** **Exercise for 4 and 12 weeks** TNF-*α* mRNA: NS TNF-*α* protein: NS	**Exercise for 4 weeks**BM: NS Mesenteric and subcutaneous FM: NS **Exercise for 12 weeks** BM: NS Mesenteric and subcutaneous FM: decrease

Miyazaki et al. [31]	Rats chow diet	Treadmill running on 5 times/week. On the first day of training, all rats ran for 30 min at 15 m/min, and then running time and velocity were extended until rats were running for 90 min at 30 m/min.	None	9 weeks	Epididymal, retroperitoneal, and inguinal WAT	**Epididymal adipocyte** Leptin mRNA: NS Adiponectin mRNA: increase (*P* < 0.05) **Retroperitoneal adipocyte** Leptin mRNA: NS Adiponectin NS **Inguinal adipocyte** Leptin mRNA: NS Adiponectin mRNA: increase (*P* < 0.05)	BM: decrease epididymal, retroperitoneal, and inguinal FM: decrease

**Table:** (b) Human studies

Citation	Subjects	Exercise program	Diet restriction	Duration of intervention	WAT used in experiment	Effects of TR on expression of adipokines in WAT	Changes of body mass (BM), body mass index (BMI), fat mass (FM), and % body fat mass (% BFM)
Christiansen et al. [32]	Obese exercise (9 m, 10 f)	Aerobic exercise for 65–75 min on 3 times/week (energy expenditure of 500–600 kcal/session)	None	12 weeks	Abdominal subcutaneous WAT	Leptin mRNA: NS TNF-*α* mRNA: NS MCP-1 mRNA: NS Adiponectin mRNA: increase (*P* < 0.01) IL-6 mRNA: NS	BM and BMI: NS Changes in body weight after intervention were 3.5%

Christiansen et al. [32]	Obese exercise + hypocaloric diet (10 m, 11 f)	Same as above	Very low energy diet (800 kcal/day) for 8 weeks followed by a weight maintenance diet for 4 weeks	12 weeks	Abdominal subcutaneous WAT	Leptin mRNA: decrease (*P* < 0.01) TNF-*α* mRNA: NS MCP-1 mRNA: NS Adiponectin mRNA: increase (*P* < 0.01) IL-6 mRNA: NS	BM and BMI: NS changes in body weight after intervention were 11.1%
	Obese hypocaloric diet (10 m, 9 f)	None	Very low energy diet (600 kcal/day) for 8 weeks followed by a weight maintenance diet for 4 weeks	12 weeks	Abdominal subcutaneous WAT	Leptin mRNA: decrease (*P* < 0.01) TNF-*α* mRNA: NS MCP-1 mRNA: NS Adiponectin mRNA: increase (*P* < 0.01) IL-6 mRNA: NS	BM and BMI: NS changes in body weight after intervention were 10.5%

Bruun et al. [33]	Obese (11 m, 12 f)	Exercise training consisted of at least 2-3 h of moderate intensity physical activity (e.g., walking, swimming, aerobics) on 5 times/week	Hypocaloric diet calculated to reduce the subject's body weight by ~1%/week	15 weeks	Abdominal subcutaneous WAT	TNF-*α* mRNA: decrease (*P* < 0.01) MCP-1 mRNA: NS Adiponectin mRNA: increase (*P* < 0.001) IL-6 mRNA: decrease (*P* < 0.05)	BM, BMI, and FM: decrease

Results are reported as mean ± SD or SE; *P* value reported for sedentary control group versus exercise trained group or pre- versus postvalues. f: female; HFD: high fat diet; m: male; NS: not significant; $\dot{\text{V}}$O_2_ max: maximal oxygen uptake; WAT: white adipose tissue.

**Table 2:** Effects of exercise training on human blood levels of leptin.

Citation	*n*, gender	Group	Exercise program	Diet restriction	Duration of intervention	Preleptin (ng/mL)	Postleptin (ng/mL)	*P* value	Changes of body mass (BM), body mass index (BMI), fat mass (FM), and % body fat mass (% BFM)
Aerobic exercise									

Houmard et al. [35]	7 m, 9 f	Younger lean	Cycle ergometer at 70–75% $\dot{\text{V}}$O_2_ max for 60 min	None	7 days	7.1 ± 1.3	7.6 ± 1.3	NS	BM: NS
	6 m, 8 f	Older subjects with relatively more adipose tissue	Same as above	None	7 days	14.2 ± 2.7	11.0 ± 1.3	NS	BM: NS

Halle et al. [36]	20 m	Obese with T2DM	Cycle ergometer for 30 min on 5 times/week at 70% HRM (1,100 kcal/wk)	Diet consisted of a 1,000-kcal diabetic diet with a carbohydrate content of ~50%, a fat content of 25%, and a protein content of 25%	4 weeks	7.9 ± 4.4	5.6 ± 3.5	P < 0.001	BMI: decrease

Ishii et al. [37]	9 m, 14 f	T2DM exercise training with diet therapy	Walking and cycle ergometer exercise at 50% of $\dot{\text{V}}$O_2_ max for 60 min on at least 5 times/week	25- to 27-kcal/kg/day diet (54% to 58% carbohydrate, 22% to 24% protein, 18% to 20% fat)	6 weeks	7.2 ± 3.6	4.6 ± 2.5	P < 0.05	BM, BMI, and % BFM: NS
	11 m, 16 f	T2DM diet therapy alone	None	Same as above	6 weeks	6.9 ± 3.4	5.6 ± 2.9	NS	BM, BMI, and % BFM: NS

Boudou et al. [38]	8 m	T2DM control	None	None	8 weeks	7.26 ± 3.85	7.40 ± 3.95	NS	BM and BMI: NS; Visceral and subcutaneous adipose tissue (cm^2^): NS
	8 m	T2DM exercise	Endurance exercise (75% VO_2_ peak, 45 min) twice a week, with intermittent exercise (five 2 min exercises at 85% VO_2_ peak separated by 3 min exercises at 50% VO_2_ peak) once a week, on a cycle ergometer	None	8 weeks	6.05 ± 4.60	5.60 ± 4.30	NS	BM and BMI: NS; Visceral and subcutaneous adipose tissue (cm^2^): decrease

Kraemer et al. [39]	14 f	Overweight control	None	None	9 weeks	33.24 ± 3.78	34.69 ± 3.14	NS	BM, BMI, and % BFM: NS
	16 f	Overweight exercise	Three-four times/week of four 20–30 min/session. Two of the exercise days consisted of step aerobics and 1-2 of the exercise days consisted of treadmill or stationary cycle exercise	None	9 weeks	28.0 ± 2.13	31.04 ± 2.71	NS	BM, BMI, and % BFM: NS

Hickey et al. [40]	9 m	Middle aged sedentary	Exercise training consists of overground and/or treadmill walking and/or running for 45 min on 4 times/week at 85% HRM	None	12 weeks			NS	BM, FM, and % FM: NS
	9 f	Middle aged sedentary	Same as above	None	12 weeks		Decrease of 17.5%	P < 0.05	BM, FM, and % FM: NS

Ozcelik et al. [41]	14 f	Obese	Cycle ergometer for approximately 45 min on 3-4 times/week. Training exercise intensity was established using the anaerobic threshold.	None	12 weeks	23.62 ± 3.5	13.13 ± 3.4	P = 0.0001	BM, BMI, and FM: decrease

Polak et al. [42]	25 f	Obese premenopausal	Aerobic exercise (aerobic exercise performed in gymnasium and cycleergometer) for 45 min on 5 times/week at 50% $\dot{\text{V}}$O_2_ max	None	12 weeks	24.3 ± 8.7	18.1 ± 8.3	P < 0.001	BM, BMI, and % BFM: decrease

Okazaki et al. [43]	15 f	Obese	Cycle ergometer or indoor walking for 30 min and low-impact aerobics for 30 min at 50% $\dot{\text{V}}$O_2_ max	Mild hypocalbolic diet	12 weeks	14.7 ± 5.3	8.9 ± 3.6	P < 0.001	BM, BMI, and FM: decrease
	26 f	Nonobese	Same as above	Same as above	12 week	7.6 ± 3.9	5.6 ± 2.2	P < 0.01	BM, BMI, and FM: decrease

Pérusse et al. [44]	51 m	Sedentary adult	The subjects worked on cycle ergometer at an intensity corresponding to 55% of $\dot{\text{V}}$O_2_ max for 30 min per session at the beginning, increasing progressively toward an intensity of 75% of $\dot{\text{V}}$O_2_ max for 50 min during the last 6 weeks of the training protocol.	None	20 weeks	4.6 ± 4.4	3.9 ± 4.2	P = 0.004	BMI: NS; FM and % BFM: decrease
	46 f		Same as above	None	20 weeks	11.9 ± 8.5	12.4 ± 8.1	NS	BMI, FM, and % BMF: NS

Kondo et al. [45]	8 f	Nonobese control	None	None	7 months	6.7 ± 1.2	6.5 ± 2.2	NS	BM, BMI: NS; FM and % BFM: decrease
	8 f	Obese	Exercise training (fast slope walking, slope jogging, dumbbells, stretching, leg cycling, and jumping rope) for 30–60 min at 60–70% HRR on 4-5 times/week	None	7 months	16.4 ± 4.6	12.3 ± 5.4	P < 0.05	BM, BMI, FM, and % BFM: decrease

Reseland et al. [46]	37 m	MS control	None	None	1 year	12.0 ± 10.1	0.5 ± 4.6 (Change)	NS	BMI, FM, and % BFM: NS
	44 m	MS diet	None	Dietary counseling	1 year	8.7 ± 4.3	−0.7 ± 3.0	P < 0.05	BMI, FM, and % BFM: decrease
	48 m	MS exercise	Endurance exercise (aerobics, circuit training, and fast walking) and jogging for 60 min on 3 times/week	None	1 year	9.8 ± 4.9	−0.4 ± 2.3	NS	BMI: NS; FM and % BFM: decrease
	57 m	MS diet + exercise	Same as above	Dietary counseling	1 year	9.1 ± 6.2	−2.2 ± 2.4	P < 0.001	BMI, FM, and % BFM: decrease

Miyatake et al. [47]	36 m	Overweight	Aerobic exercise (walking, aerobic dance, and swimming) and resistance training (leg extension and leg flexion) for 90 min at 50–65% HRM	None	1 year	6.7 ± 4.0	5.1 ± 3.1	P < 0.01	BM, BMI, FM, and % BFM: decrease

Hsieh and Wang [48]	22 m, 30 f	Younger T2DM	Endurance exercise for 20 min at 50–74% HRM	Subjects were prescribed a diet with 500 kcal/day deficit.	1 year	17.62 ± 3.18	14.00 ± 3.16	P = 0.03	BMI, and % BFM: decrease
	20 m, 30 f	Older T2DM	Same as above	Same as above	1 year	17.81 ± 2.15	12.63 ± 2.09	P = 0.02	BMI, and % BFM: decrease

Resistance exercise									

Ryan et al. [49]	8 f	Nonobese postmenopausal women RT	Three exercise sessions/week on pneumatic variable resistance machines	None	16 weeks	14.6 ± 3.3	14.8 ± 3.0	NS	BM, BMI, FM, and % BFM: NS
	7 f	Obese postmenopausal women RT + WL	Same as above	Dietary counseling and energy restriction (hypocaloric diets)	16 weeks	22.9 ± 3.9	14.6 ± 2.6	*P* < 0.01	BM, BMI, FM, and % BFM: decrease

Fatouros et al. [50]	10 m	Overweight elderly control	None	None	24 weeks	9.5 ± 0.8	9.4 ± 0.7	NS	BM and BMI: NS
	14 m	Overweight elderly low-intensity RT	RT for approximately 60 min on 3 times/week at 45–50% of 1RM	None	24 weeks	9.1 ± 0.7	8.8 ± 0.7	*P* < 0.05	BM: NS; BMI: decrease
	12 m	Overweight elderly moderate- intensity RT	RT for approximately 60 min on 3 times/week at 60–65% of 1RM	None	24 weeks	8.9 ± 0.6	8.7 ± 0.4	*P* < 0.05	BM: NS; BMI: decrease
	14 m	Overweight elderly high- intensity RT	RT for approximately 60 min on 3 times/week at 80–85% of 1RM	None	24 weeks	9.7 ± 0.6	7.8 ± 0.6	*P* < 0.05	BM: NS; BMI: decrease

Results are reported as mean ± SD or SE; *P* value reported for pre- versus postvalues. f: female; HRM: heart rate maximum; m: male; NS: not significant; RM: repetition maximum; RT: resistance training; T2DM: type 2 diabetes; $\dot{\text{V}}$O_2_ max: maximal oxygen uptake; WL: weight loss.

**Table:** (a) TNF-*α*

Citation	*n*, gender	Group	Exercise program	Diet restriction	Duration of intervention	Pre-TNF-*α* (pg/mL)	Post-TNF-*α*(pg/mL)	*P* value	Changes of body mass (BM), body mass index (BMI), fat mass (FM), and % body fat mass (% BFM)
Katsuki et al. [59]	11 m, 1 f	Nonobese NIDDM	Walking about 15,000 steps daily	Dietary treatment (1400–1720 kcal/day with a diet consisting of 20 energy percent (en%) protein, 25 en% fat, and 55 en% carbohydrates	4 weeks			NS	BMI and visceral adipose tissue area (cm^2^): decrease; subcutaneous adipose tissue (cm^2^): NS
	11 m, 1 f	Obese-NIDDM	Same as above	Same as above	4 weeks		Decrease	*P* < 0.01	BMI, visceral and subcutaneous adipose tissue area (cm^2^): decrease;

Stŗczkowski et al. [60]	8 f	Obese with normal glucose tolerance	Cycle ergometer for 30 min on 5 times/week at 70% HRM	None	12 weeks	3.88 ± 0.49	3.27 ± 0.54	*P* < 0.05	BM, BMI, FM, and % BFM: decrease
	8 f	Obese with impaired glucose tolerance	Same as above	None	12 weeks	6.59 ± 2.31	5.15 ± 1.19	*P* < 0.05	BM, BMI, and % BFM: decrease

Polak et al. [42]	25 f	Obese premenopausal	Aerobic exercise (aerobic exercise performed in gymnasium and cycleergometer) for 45 min on 5 times/week at 50% $\dot{\text{V}}$O_2_ max	None	12 week	6.1 ± 7.6	4.8 ± 4.5	*P* = 0.08	BM, BMI, and % BFM: decrease

Bruun et al. [33]	11 m, 12 f	Obese	Exercise training consisted of at least 2-3 h of moderate intensity physical activity (e.g., walking, swimming, aerobics) on 5 times/week	Hypocaloric diet calculated to reduce the subject's body weight by ~1%/week	15 weeks	1.0 ± 0.08	1.0 ± 0.2	NS	BM, BMI, and FM: decrease

Kondo et al. [45]	8 f	Nonobese control	None	None	7 months	2.3 ± 0.9	2.1 ± 1.4	NS	BM, BMI: NS; FM and % BFM: decrease
	8 f	Obese	Exercise training (fast slope walking, slope jogging, dumbbells, stretching, leg cycling, and jumping rope) for 30–60 min at 60–70% HRM on 4-5 times/week	None	7 months	7.6 ± 2.3	4.8 ± 1.2	*P* < 0.01	BM, BMI, FM, and % BFM: decrease

Horne et al. [61]	7 m	Healthy endurance training	Cycle ergometers 2 times/week for 30 min and progressed to 42 min (a 4-min increase every 4 weeks) at a power output equivalent to that at ventilation threshold	None	12 weeks	5.7 ± 4.4	6.0 ± 4.0 (6 weeks) 5.9 ± 2.7 (12 weeks)	NS	
	4 f		Same as above	None	12 weeks	5.6 ± 3.7	37.8 ± 24.7^a^ (6 weeks) 17.6 ± 6.4^b^ (12 weeks)	^ a^ *P* < 0.05 (versus pre) ^b^ *P* < 0.05 (versus pre and 6 weeks)	
	7 m	Healthy resistance training	Resistance training by using machine on 3 times/week	None	12 weeks	9.5 ± 3.0	10.8 ± 4.6 (6 week) 5.8 ± 2.9 (12 week)	NS	
	4 f		Same as above	None	12 weeks	2.8 ± 2.0	6.6 ± 4.08 (6 weeks) 0.3 ± 0.5 (12 weeks)	NS	
	8 m	Healthy endurance and resistance training	Combination of above endurance and resistance training	None	12 weeks	2.3 ± 1.9	4.7 ± 0.5 (6 weeks) 5.6 ± 2.9 (12 weeks)	NS	
	5 f		Same as above	None	12 weeks	4.5 ± 2.0	8.0 ± 4.0 (6 weeks) 4.5 ± 0.5 (12 weeks)	NS	

Kohut et al. [62]	40	Overweight aerobic exercise with or without *β*-blocker treatment	Aerobic exercise for 45 min on 3 times/week	None	10 months		Decrease	Main effect of time, *P* = 0.001	BMI: NS
	47	Over weight flexibility/strength exercise with or without *β*-blocker treatment	Flexibility/strength exercise for 45 min on 3 times/week	None	10 months		Decrease	Main effect of time, *P* = 0.001	BMI: NS

Nicklas et al. [63]	Base line: 70 6 months: 63: 18 months: 60	Overweight or obese older control	None	None	18 months	3.8 ± 7.5	Changes −0.74 ± 3.7 (6 months) −0.77 ± 3.7 (18 months)	NS	BM: NS
	Base line: 67 6 months: 58: 18 months: 53	Overweight or obese exercise	Exercise program consisted of an aerobic phase (15 min), a resistance-training phase (15 min), a second aerobic phase (15 min), and a cool-down phase (15 min) on 3 times/week.	None	18 months	3.4 ± 0.8	Changes −0.69 ± 5.8 (6 months) 0.28 ± 6.3 (18 months)	NS	BM: NS
	Base line: 71 6 months: 63: 18 months: 53	Overweight or obese dietary WL	None	Counseling to decrease their energy intake by 500 kcal/day	18 months	2.5 ± 1.8	Changes −0.23 ± 1.8 (6 months) 0.64 ± 5.9 (18 months)	NS	BM: decrease
	Base line: 64 6 months: 58 18 months: 53	Overweight or obese exercise + dietary WL	Exercise program consisted of an aerobic phase (15 min), a resistance-training phase (15 min), a second aerobic phase (15 min), and a cool-down phase (15 min) on 3 times/week.	Same as above	18 months	3.4 ± 6.4	Changes −0.46 ± 3.7 (6 months) −0.72 ± 4.6 (18 months)	NS	BM: decrease

**Table:** (b) MCP-1

Citation	*n*, gender	Group	Exercise program	Diet restriction	Duration of intervention	Pre-MCP-1 (pg/mL)	Post-MCP-1(pg/mL)	*P* value	Changes of BM, BMI, FM, and % BFM
Trøseid et al. [64]	14	MS with or without administration of pravastatin control	None	None	12 weeks		−2.0 (the changes from baseline in plasma levels of MCP-1)	NS	BMI: NS
	18	MS with or without administration of pravastatin exercise	The duration of each workout was 45–60 min. Approximately 40% of the scheduled workout was walking/jogging/cycling and 60% was strength training. The strength training was performed in cycles with 15–20 repetitions per cycle, and large muscle groups such as thighs, back, and abdomen were trained.	None	12 weeks		−50	*P* < 0.01	BMI: decrease

Christiansen et al. [32]	9 m, 10 f	Obese exercise	Aerobic exercise for 65–75 min on 3 times/week (energy expenditure of 500–600 kcal/session)	None	12 weeks		Decreasing trend (Relative changes)	*P* = 0.06	BM and BMI: NS Changes in body weight after intervention were 3.5%
	10 m, 11 f	Obese exercise + hypocaloric diet	Same as above	Very low energy diet (800 kcal/day) for 8 weeks followed by a weight maintenance diet for 4 weeks	12 weeks		Decrease	*P* < 0.05	BM and BMI: NS Changes in body weight after intervention were 11.1%
	10 m, 9 f	Obese hypocaloric diet	None	Very low energy diet (600 kcal/day) for 8 weeks followed by a weight maintenance diet for 4 weeks	12 weeks		Decrease	*P* < 0.05	BM and BMI: NS Changes in body weight after intervention were 10.5%

Bruun et al. [33]	11 m, 12 f	Obese	Exercise training consisted of at least 2-3 h of moderate intensity physical activity (e.g., walking, swimming, aerobics) on 5 times/week	Hypocaloric diet calculated to reduce the subject's body weight by ~1%/week	15 weeks	141.2 ± 8.3	122.0 ± 6.3	*P* < 0.01	BM, BMI, and FM: decrease

Results are reported as mean ± SD or SE; *P* value reported for pre- versus post values. f: female; HRM: heart rate maximum; m: male; MS: metabolic syndrome; NIDDM: noninsulin dependent diabetes mellitus; NS: not significant; RM: repetition maximum; $\dot{\text{V}}$O_2_ max: maximal oxygen uptake; WL: weight loss.

**Table 4:** Effects of exercise training on human blood levels of adiponectin.

Citation	*n*, gender	Group	Exercise program	Diet restriction	Duration of intervention	Preadiponectin (*μ*g/mL)	Postadiponectin (*μ*g/mL)	*P* value	Changes of body mass (BM), body mass index (BMI), fat mass (FM), and % body fat mass (% BFM)
Aerobic exercise									

Blüher et al. [65]	9 m, 11 f	Normal glucose tolerance	Exercise training consisted of 20 min of warming and cool-down periods, 20 min of running or biking, and 20 min of swimming on 3 times/week	None	4 weeks	8.7 ± 0.6	9.8 ± 0.6	*P* < 0.01	BM, BMI, and % BFM: decrease
	9 m, 11 f	Impaired glucose tolerance	Same as above	None	4 weeks	3.4 ± 0.26	6.7 ± 0.7	*P* < 0.001	BM, BMI, and % BFM: decrease
	11 m, 9 f	T2DM	Same as above	None	4 weeks	3.5 ± 0.4	6.5 ± 0.6	*P* < 0.001	BM, BMI, and % BFM: decrease

Oberbach et al. [66]	9 m, 11 f	Normal glucose tolerance	Exercise training consisted of 20 min warming and cool-down periods, 20 min of running or biking, and 20 min of powertraining	None	4 weeks			NS	BM, BMI, and % BFM: decrease
	9 m, 11 f	Impaired glucose tolerance	Same as above	None	4 weeks		Increase	*P* < 0.001	BM, BMI, and % BFM: decrease
	11 m, 9 f	T2DM	Same as above	None	4 weeks		Increase	*P* < 0.001	BM, BMI, and % BFM: decrease

Boudou et al. [38]	8 m	T2DM control	None	None	8 weeks	7.30 ± 2.55	7.05 ± 2.10	NS	BM and BMI: NS; visceral and subcutaneous adipose tissue area (cm^2^): NS
	8 m	T2DM exercise	Endurance exercise (75% VO_2_ peak, 45 min) twice a week, with intermittent exercise (five 2 min exercises at 85% VO_2_ peak separated by 3 min exercises at 50% VO_2_ peak) once a week, on a cycle ergometer	None	8 weeks	6.30 ± 2.75	6.00 ± 3.50	NS	BM and BMI: NS; visceral and subcutaneous adipose tissue area (cm^2^): decrease

O'Leary et al. [67]	4 m, 7 f	Older insulin-resistant exercise + hypocaloric diet	Aerobic exercise for 60 min at 80–85% HRM on 5 times/week	Diet with total energy content calculated to reduce body weight by 10–15% (~1,300 kcal/day).	12 weeks	7.6 ± 0.9	6.6 ± 1.0	NS	BM, BMI, and FM: decrease
	3 m, 7 f	Older insulin-resistant exercise + eucaloric diet	Same as above	Weight maintenance diet that consisted of their usual food consumption (~1,800 kcal/day)		7.7 ± 1.2	6.8 ± 1.6	NS	BM, BMI, and FM: decrease

Polak et al. [42]	25 f	Obese premenopausal	Aerobic exercise (aerobic exercise performed in gymnasium and cycleergometer) for 45 min on 5 times/week at 50% $\dot{\text{V}}$O_2_ max	None	12 weeks	10.9 ± 6.1	10.0 ± 4.4	NS	BM, BMI, and % BFM: decrease

Nassis et al. [68]	21 f	Overweight/obese girls	Aerobic training for 40 min (10 min of warm up, 25 min of physical training games, and 5 minutes of cool down) on 3 times/week	None	12 weeks	9.57 ± 3.01	9.08 ± 2.32	NS	BM, BMI, and % BFM: NS

Christiansen et al. [32]	9 m, 10 f	Obese exercise	Aerobic exercise for 65–75 min on 3 times/week (energy expenditure of 500–600 kcal/session)	None	12 weeks			NS	BM and BMI: NS Changes in body weight after intervention were 3.5%
	10 m, 11 f	Obese exercise + hypocaloric diet	Same as above	Very low energy diet (800 kcal/day) for 8 weeks followed by a weight maintenance diet for 4 weeks	12 weeks		Increase	*P* < 0.05	BM and BMI: NS Changes in body weight after intervention were 11.1%
	10 m, 9 f	Obese hypocaloric diet	None	Very low energy diet (600 kcal/day) for 8 weeks followed by a weight maintenance diet for 4 weeks	12 weeks		Increase	*P* < 0.05	BM and BMI: NS Changes in body weight after intervention were 10.5%

Bruun et al. [33]	11 m, 12 f	Obese	Exercise training consisted of at least 2-3 h of moderate intensity physical activity (e.g., walking, swimming, aerobics) on 5 times/week	Hypocaloric diet calculated to reduce the subject's body weight by ~1%/week	15 weeks	5.2 ± 0.6	6.9 ± 0.5	*P* < 0.001	BM, BMI, and FM: decrease

Hulver et al. [69]	8 m, 3 f	Nonobese exercise	Treadmill walking/running, stair climbing, and cycling for 45 min at 65–80% $\dot{\text{V}}$O_2_ max on 4 times/week	None	6 months	6.3 ± 1.5	6.6 ± 1.8	NS	BM, BMI, and FM: NS
	3 m, 11 f	Obese weight loss	None	Gastric bypass surgery	6 months	4.4 ± 0.8	13.6 ± 2.2	*P* < 0.05	BM and BMI: decrease

Kondo et al. [45]	8 f	Nonobese control	None	None	7 months	8.3 ± 1.5	8.2 ± 2.3	NS	BM, BMI: NS; FM and % BFM: decrease
	8 f	Obese	Exercise training (fast slope walking, slope jogging, dumbbells, stretching, leg cycling, jumping rope) for 30–60 min at 60–70% HRM on 4-5 times/week	None	7 months	2.4 ± 1.3	4.2 ± 1.2	*P* < 0.01	BM, BMI, FM, and % BFM: decrease

Hsieh and Wang [48]	22 m, 30 f	Younger T2DM	Endurance exercise for 20 min at 50–74% HRM	Subjects were prescribed a diet with 500 kcal/day deficit.	1 year	4.13 ± 0.88	5.47 ± 0.59	*P* = 0.04	BMI, and % BFM: decrease
	20 m, 30 f	Older T2DM	Same as above	Same as above	1 year	4.26 ± 0.97	6.56 ± 0.86	*P* = 0.03	BMI, and % BFM: decrease

Resistance exercise									

Fatouros et al. [50]	10 m	Overweight elderly control	None	None	24 weeks	7.22 ± 2.7	7.84 ± 3.5	NS	BM and BMI: NS
	14 m	Overweight elderly low- intensity RT	Resistance training for approximately 60 min on 3 times/week at 45–50% of 1RM	None	24 weeks	7.45 ± 2.3	8.48 ± 2.2	NS	BM: NS; BMI: decrease
	12 m	Overweight elderly moderate- intensity RT	Resistance training for approximately 60 min on 3 times/week at 60–65% of 1RM	None	24 weeks	7.79 ± 1.4	9.48 ± 1.1	*P* < 0.05	BM: NS; BMI: decrease
	14 m	Overweight elderly high- intensity RT	Resistance training for approximately 60 min on 3 times/week at 80–85% of 1RM	None	24 weeks	7.04 ± 1.6	11.36 ± 1.6	*P* < 0.05	BM: NS; BMI: decrease

Results are reported as mean ± SD or SE; *P* value reported for pre- versus post values. f: female; HRM: heart rate maximum; m: male; NS: not significant; RM: repetition maximum; RT: resistance training T2DM: type 2 diabetes; $\dot{\text{V}}$O_2_ max: maximal oxygen uptake.

**Table 5:** Effects of exercise training on human blood levels of IL-6.

Citation	*n*, gender	Group	Exercise program	Diet restriction	Duration of intervention	Pre-IL-6 (pg/mL)	Post-IL-6(pg/mL)	*P* value	Changes of body mass (BM), body mass index (BMI), fat mass (FM), and % body fat mass (% BFM)
Oberbach et al. [66]	9 m, 11 f	Normal glucose tolerance	Exercise training consisted of 20 min warming and cool-down periods, 20 min of running or biking, and 20 min of powertraining	None	4 weeks			NS	BM, BMI, and %BFM: decrease
	9 m, 11 f	Impaired glucose tolerance	Same as above	None	4 weeks			NS	BM, BMI, and % BFM: decrease
	11 m, 9 f	T2DM	Same as above	None	4 weeks			NS	BM, BMI, and % BFM: decrease

Polak et al. [42]	25 f	Obese premenopausal	Aerobic exercise (aerobic exercise performed in gymnasium and cycleergometer) for 45 min on 5 times/week at 50% $\dot{\text{V}}$O_2_ max	None	12 weeks	3.1 ± 3.7	1.4 ± 1.5	NS	BM, BMI, and % BFM: decrease

Nassis et al. [68]	21 f	Overweight/obese girls	Aerobic training for 40 min (10 min of warm up, 25 min of physical training games, and 5 minutes of cool down) on 3 times/week	None	12 weeks	1.67 ± 1.29	1.65 ± 1.25	NS	BM, BMI, and % BFM: NS

Bruun et al. [33]	11 m, 12 f	Obese	Exercise training consisted of at least 2-3 h of moderate intensity physical activity (e.g., walking, swimming, aerobics) on 5 times/week	Hypocaloric diet calculated to reduce the subject's body weight by ~1%/week	15 weeks	4.6 ± 0.6	3.4 ± 0.6	*P* < 0.01	BM, BMI, and FM: decrease

Kohut et al. [62]	40	Overweight aerobic exercise with or without *β*-blocker treatment	Aerobic exercise for 45 min on 3 times/week	None	10 months		Decrease	Significant treatment × time interaction. *P* < 0.05	BMI: NS
	47	Overweight flexibility/strength exercise with or without *β*-blocker treatment	Flexibility/strength exercise for 45 min on 3 times/week	None	10 months			NS	BMI: NS

Nicklas et al. [63]	Base line: 70 6 months: 63: 18 months: 60	Overweight or obese older control	None	None	18 months	4.7 ± 3.2	Changes 0.19 ± 2.8 (6 months) 0.27 ± 2.8 (18 months)	NS	BM: NS
	Base line: 67 6 months: 58: 18 months: 53	Overweight or obese exercise	Exercise program consisted of an aerobic phase (15 min), a resistance-training phase (15 min), a second aerobic phase (15 min), and a cool-down phase (15 min) on 3 times/week.	None	18 months	4.4 ± 3.1	Changes 0.15 ± 1.8 (6 months) 0.02 ± 2.4 (18 months)	NS	BM: NS
	Base line: 71 6 months: 63: 18 months: 53	Overweight or obese dietary WL	None	Counseling to decrease their energy intake by 500 kcal/day	18 months	4.7 ± 3.4	Changes −0.51 ± 2.1 (6 months) −0.71 ± 2.4 (18 months)	Main effect of WL, *P* = 0.009	BM: decrease
	Base line: 64 6 months: 58 18 months: 53	Overweight or obese exercise + dietary WL	Exercise program consisted of an aerobic phase (15 min), a resistance-training phase (15 min), a second aerobic phase (15 min), and a cool-down phase (15 min) on 3 times/week.	Same as above	18 months	4.9 ± 3.0	Changes −0.35 ± 2.15 (6 months) −0.35 ± 1.8 (18 months)	Main effect of WL, *P* = 0.009	BM: decrease

Results are reported as mean ± SD or SE; *P* value reported for pre- versus post values. f: female; HRM: heart rate maximum; m: male; NS: not significant; WL: weight loss.
